# lncTUG1/miR-144-3p affect the radiosensitivity of esophageal squamous cell carcinoma by competitively regulating c-MET

**DOI:** 10.1186/s13046-019-1519-y

**Published:** 2020-01-09

**Authors:** Pan Wang, Zhuanbo Yang, Ting Ye, Fei Shao, Jiagen Li, Nan Sun, Jie He

**Affiliations:** 1 Department of Thoracic Surgery, National Cancer Center/National Clinical Research Center for Cancer/Cancer Hospital, Chinese Academy of Medical Sciences and Peking Union Medical College, No.17 Panjiayuannanli, Beijing, 100021 China; 2 Department of Radiation Oncology, National Cancer Center/National Clinical Research Center for Cancer/Cancer Hospital, Chinese Academy of Medical Sciences and Peking Union Medical College, Beijing, 100021 China; 3 Cancer Institute, The Affiliated Hospital of Qingdao University, Qingdao, 266071 Shandong China; 4 Qingdao Cancer Institute, Qingdao, 266071 Shandong China

**Keywords:** lncTUG1, miR-144-3p, MET, Radiosensitivity, Esophageal squamous cell carcinoma

## Abstract

**Background:**

Long noncoding RNAs (lncRNAs) are involved in the progression of various cancers and affect the response to radiotherapy. This study focused on clarifying the underlying mechanism by which lncTUG1 affects the radiosensitivity of esophageal squamous cell carcinoma (ESCC).

**Methods:**

lncTUG1, miR-144-3p and MET expression levels were detected in ESCC tissues and cells by qRT-PCR. Western blotting was used to examine the protein levels of MET, p-AKT and EGFR. The dual-luciferase reporter system and RNA immunoprecipitation (RIP) assays were used to confirm the interaction between lncTUG1 and miR-144-3p or miR-144-3p and MET. MTT, colony formation and flow cytometry assays were applied to examine the behavioral changes in EC9706 and KYSE30 cells.

**Results:**

lncTUG1 was upregulated in ESCC cells and tissues, and lncTUG1 expression was associated with an advanced pathological stage. The bioinformatics analysis revealed that lncTUG1 could specifically bind to miR-144-3p, which was downregulated in ESCC. There was a negative correlation between lncTUG1 and miR-144-3p. LncTUG1 inhibition retarded proliferation and colony formation and induced apoptosis in ESCC cells. Moreover, lncTUG1 knockdown dramatically improved the effect of radiotherapy on ESCC development both in vivo and in vitro. Furthermore, MET was revealed as a downstream target of miR-144-3p and is downregulated by it. LncTUG1 promoted the progression of ESCC and elevated radiotherapy resistance in ESCC cells, accompanied by a high level of MET expression. Moreover, we found that knockdown of lncTUG1 enhanced the radiosensitivity of ESCC cells via the p-AKT signaling pathway.

**Conclusion:**

Our results indicate that lncTUG1 enhances the radiotherapy resistance of ESCC by lowering the miR-144-3p level and modulating the MET/EGFR/AKT axis.

## Background

Esophageal carcinoma ranks 9th among the most lethal cancers and widely exists in the world. According to statistics, esophageal carcinoma is responsible for hundreds of thousands of deaths [1, 2]. Esophageal squamous cell carcinoma (ESCC) is the predominant histological subtype, accounting for 90% of all cases. As a high aggressive malignancy, ESCC always accompanies a miserable clinical outcome [3]. Despite noteworthy advances in cancer diagnosis and therapy, the clinical outlook of ESCC patients remains dismal, with a five-year survival rate of less than 30% [4, 5]. To date, traditional surgery remains the preferred treatment for patients with early ESCC, but for patients with advanced ESCC, chemotherapy or radiotherapy is used [6]. However, there are quite a few patients who do not benefit from single radiotherapy or obtain an ideal response [7]. Thus, there is an urgent need to find a potential biological marker to indicate radiosensitivity and guide radiotherapy in ESCC patients.

Recently, long noncoding RNAs (lncRNAs) have been described as noncoding RNAs that participate in many cancers and affect the progression of tumors [8, 9]. lncRNAs, long RNAs > 200 nucleotides (nt) in length without any detectable open reading frames, regulate distinct biological processes in cancer cells by sponging microRNAs (miRNAs) or impacting the functions of related proteins [10]. Previous studies have shown that a high level of lncTUG1 accelerates cell growth by silencing KLF2 in hepatocellular carcinoma [11]. Similar to oncogenic factors, the role of lncTUG1 in ESCC is to promote the proliferation and migration of ESCC [12]. Moreover, lncRNAs have been found to influence radiosensitivity by various mechanisms, including DNA damage repair, epithelial-mesenchymal transition (EMT), apoptosis, and autophagy [13]. For instance, lncFAM201 regulates the radiosensitivity of non-small cell lung cancer (NSCLC) by the EGFR/miR-370 axis [14]. However, whether lncTUG1 is involved in the regulation of the radiosensitivity of ESCC remains uncharacterized.

Numerous miRNAs affect many human diseases, especially cancers [15]. miRNAs are noncoding RNAs 20–25 nt in length that bind to the 3′ untranslated region (3′-UTR) of a specific mRNA, resulting in degradation of the target mRNA or repression of the mRNA expression [16, 17]. Many miRNAs have been verified to be related to anticancer treatments, including radiotherapy [18]. For instance, miR-145 regulates radiotherapy resistance by affecting the P53 signaling pathway in colorectal cancer [19]. At present, the important role of miR-144-3p as a tumor suppressor in cancer is uncovered [20, 21]; however, whether miR-144-3p acts as a radiosensitivity-related factor in ESCC cell lines and tissues needs to be investigated.

C-MET is a receptor tyrosine kinase and activates a wide range of different cellular signaling pathways after binding to its ligand, hepatocyte growth factor [22]. MET is always associated with EGFR and can upregulate EGFR to increase the phosphorylation of AKT (p-AKT) [23]. As a key factor related to radiosensitivity, a high level of AKT phosphorylation usually reflects a resistance effect on cancer radiotherapy [24, 25]. Thus, it is critical to reduce the p-AKT level to improve the benefits of cancer radiation therapy.

The aim of this study was to discover mechanisms that can enhance the response of ESCC to radiotherapy. Through bioinformatics analysis, we found that the lncRNA TUG1 may be involved in regulating the radiosensitivity of ESCC, and the role of lncTUG1 in ESCC was subsequently examined. These findings suggest that lncTUG1 enhances the radiotherapy resistance of ESCC by lowering the miR-144-3p level and modulating the MET/EGFR/AKT axis. Therefore, lncTUG1 provides a new possible theoretical basis for radiotherapy in ESCC and has become a potential therapeutic target.

## Methods

### Clinical samples

A total of 50 paired tumor and adjacent normal tissues were retrospectively collected from 50 patients with ESCC. All of the patients had primary, nondistant metastatic ESCC and had undergone complete surgical resection (esophagectomy) at the Cancer Hospital of the Chinese Academy of Medical Sciences (CAMS) between December 2014 and December 2018 after providing informed written consent and agreement. None of the patients received chemo- or radiotherapy prior to surgery. According to the National Comprehensive Cancer Network esophageal cancer guidelines, the normal tissues were at least 5 cm away from the primary lesions. All samples were stored at − 80 °C before further processing. This study was approved by the Medical Ethics Committee of the Cancer Hospital of the CAMS. The clinical characteristics of the patients are shown in Table 1.

**Table 1:** The relationships between TUG1 expression level and clinicopathological characteristics of patients with ESCC

Characteristics	Expression of		*P* value
	Low(*n* = 25)	High(*n* = 25)	
Sex			0.248
Male	17	13	
Female	8	12	
Age			0.009*
≤ 60	14	5	
> 60	11	20	
Tumor size (cm)			0.001*
≤ 5	15	6	
> 5	10	19	
Lymph node metastasis			0.045*
Yes	11	18	
No	14	7	
Pathological Staging			0.152
I + II	13	8	
III + IV	12	17	
Smoking status			0.239
Ever/current	14	18	
Never	11	7	
Alcohol consumption			0.771
Ever/current	16	15	
Never	9	10	

Low/high by the sample mean. Pearson χ2 test. *P < 0.05 was considered statistically significant

### Bioinformatics analysis

Radiation sensitive and resistant samples were retrieved from Gene Expression Omnibus (GEO) repository (GSE61816 and GSE61772). Probes were annotated by the platform information stored in GEO. For gene with multiple probes, the expression value was calculated by averaging the expression values of its probes. To make data from different dastset comparable, the ComBat algorithm implemented in R package *sva* were used to adjust the batch effects and the batch were set as the different GEO series. R package *limma* was used to identify the differential expressed genes (DEGs). The design model were generated by “model.matrix(~ 0+ Resistance/Sensitive)”.

### Cell culture

Human esophageal epithelial cells (Het-1A) and ESCC cell lines (TE-13, KYSE140, EC9706, and KYSE30) were purchased from the Cell Bank of Type Culture Collection of Chinese Academy of Sciences (Shanghai, China) and cultured in RPMI 1640 medium supplemented with 10% fetal bovine serum (Gibco, USA) in a 37 °C incubator with 5% CO_2_.

### Quantitative real-time PCR (qRT-PCR)

Total RNA was extracted with TRIzol reagent (Invitrogen, Carlsbad, CA, USA). cDNAs were synthesized with a reverse transcription kit (Invitrogen). qRT-PCR analysis was performed with SYBR Premix Ex Taq II (TaKaRa, Dalian, China). For mRNA and miRNA, GAPDH and U6 were used as internal controls, respectively. The primers are shown in Table 2.

**Table 2:** The sequences of specific primers

Gene name	Primer sequence (5′ to3’)
lncTUG1	Forward: 5′-CTGAAGAAAGGCAACATC-3’
	Reverse: 5′-GTAGGCTACTACAGGATTTG-3’
miR-144-3p	Forward: 5′- CCCTACAGTATAGATGATG −3’
	Reverse: 5′-TGCAGGGTCCGAGGT-3’
c-Met	Forward: 5′-CATGCCGACAAGTGCAGTA-3’
	Reverse: 5′-TCTTGCCATCATTGTCCAAC-3’
GAPDH	Forward: 5′-ATCCACGGGAGAGCGACAT-3’
	Reverse: 5′-CAGCTGCTTGTAAAGTGGAC-3’
U6	Forward: 5′-ACAGATCTGTCGGTGTGGCAC-3’
	Reverse: 5′-GGCCCCGGATTATCCGACATTC-3’

### Cell transfection

After reaching 40–50% confluence, cells were transfected with a small interfering RNA (siRNA) targeting TUG1 (si-TUG1), a miR-144-3p mimic, a miR-144-3p inhibitor, si-MET, LV-TUG1 and a nonspecific control (Invitrogen, Shanghai, China) by using Lipofectamine 3000 (Invitrogen, USA).

### Dual-luciferase reporter assays

Luciferase reporter gene vectors (pRL-TK, Promega) containing wild type (WT) or mutant (Mut) lncTUG1 and the 3′-UTR of WT or Mut MET were transfected into HEK293T cells. The miR-144-3p mimic, miR-144-3p inhibitor or negative control (NC) was cotransfected with reporter plasmids for 48 h. Relative luciferase activity was determined using a Dual-Luciferase Reporter Assay System (Promega).

### Cell viability assays

A total of 5000 cells were seeded into a 96-well plate for 24 h, and then cells were exposed to 2 Gy radiation (once). After radiotherapy, cell viability was evaluated by the MTT assay at 0, 24, 48, 72 and 96 h. A range of radiation doses (0, 2, 4, 6 and 8 Gy) was applied in a dose-dependent experiment.

### Colony formation assays

Five hundred cells were seeded into a 6-well plate with or without 2 Gy radiation. After two weeks, the cells were fixed and stained with 0.1% crystal violet solution. The numbers of colonies were counted under an inverted microscope.

### Flow cytometry

EC9706 and KYSE30 cells were harvested at 48 h posttransfection. An Annexin V-FITC/PI Apoptosis Detection Kit (Sigma-Aldrich, St. Louis, MO, USA) was utilized to detect cell apoptosis according to the manufacturer’s instructions, and the percentage of apoptotic cells was calculated using a Beckman Coulter FACS flow cytometer (Beckman Coulter).

### Western blot analysis

The cells were lysed in RIPA buffer (Sigma-Aldrich). After centrifugation, the protein was extracted, and the concentration was quantified using a BCA assay (Pierce, Rockford, IL, USA). Then, protein samples were separated by 10% SDS-PAGE and transferred onto polyvinylidene fluoride (PVDF) membranes (Amersham Pharmacia, Little Chalfont, UK). The primary antibodies used were anti-c-MET (1:1000, Thermo Fisher Scientific), anti-EGFR (1:2500, Invitrogen), anti-t-AKT (1:2000, Cell Signaling), anti-p-AKT (1:500, Invitrogen), and anti-GAPDH (1:1000, Invitrogen), and a secondary horseradish peroxidase (HRP)-conjugated antibody (Invitrogen) was used. GAPDH was chosen as the internal loading control.

### RNA immunoprecipitation (RIP) assays

A Magna RIP™ RNA-Binding Protein Immunoprecipitation Kit (Millipore, USA) was used for RIP experiments according to the manufacturer’s instructions. The TUG1 level was detected by qRT-PCR.

### Xenograft mouse model

Twenty male BALB/c nude mice (age, 6 weeks; sex, male; weight, 20 g) were obtained by the Cancer Hospital of the CAMS and maintained in a pathogen-free animal facility at 24 °C with access to distilled food and water. A total of 3 × 10^6^ transfected (LV-NC or LV-TUG1) KYSE30 cells were subcutaneously injected into six-week-old male nude mice (*n* = 5 per group). The mice were given radiation (2 Gy) for 5 consecutive days when the tumors reached an average volume of approximately 100 mm^3^. Tumor volume was measured every three days according to the following formula: volume = 1/2 × length × width^2^. All animal procedures were performed following approval from the Animal Care and Use Committee of the Cancer Hospital of the CAMS.

### Immunohistochemistry

All tissues were cut into 4-μm sections. The sections were incubated with an anti-Ki67 antibody (1:200, Abcam, Cambridge, UK), MET antibody (1:200, GeneTex, GTX50668) and p-AKT antibody (1:200, GeneTex, GTX128414) at 4 °C overnight. Then, biotinylated secondary antibodies were incubated for 1 h at room temperature and visualized with diaminobenzidine substrate (Sigma-Aldrich, St. Louis, MO, USA). Immunohistochemistry (IHC) images were taken using an Olympus microscope.

### Statistical analysis

Statistical analysis was performed using SPSS 19.0 software (SPSS, Chicago, IL, USA). The data are expressed as the mean ± standard deviation (SD). Differences between groups were evaluated by Student’s t-test or one-way analysis of variance (ANOVA). *P* < 0.05 indicated statistical significance.

## Results

### Bioinformatics analysis shows that lncTUG1 might participate in ESCC

**Fig. 1:**
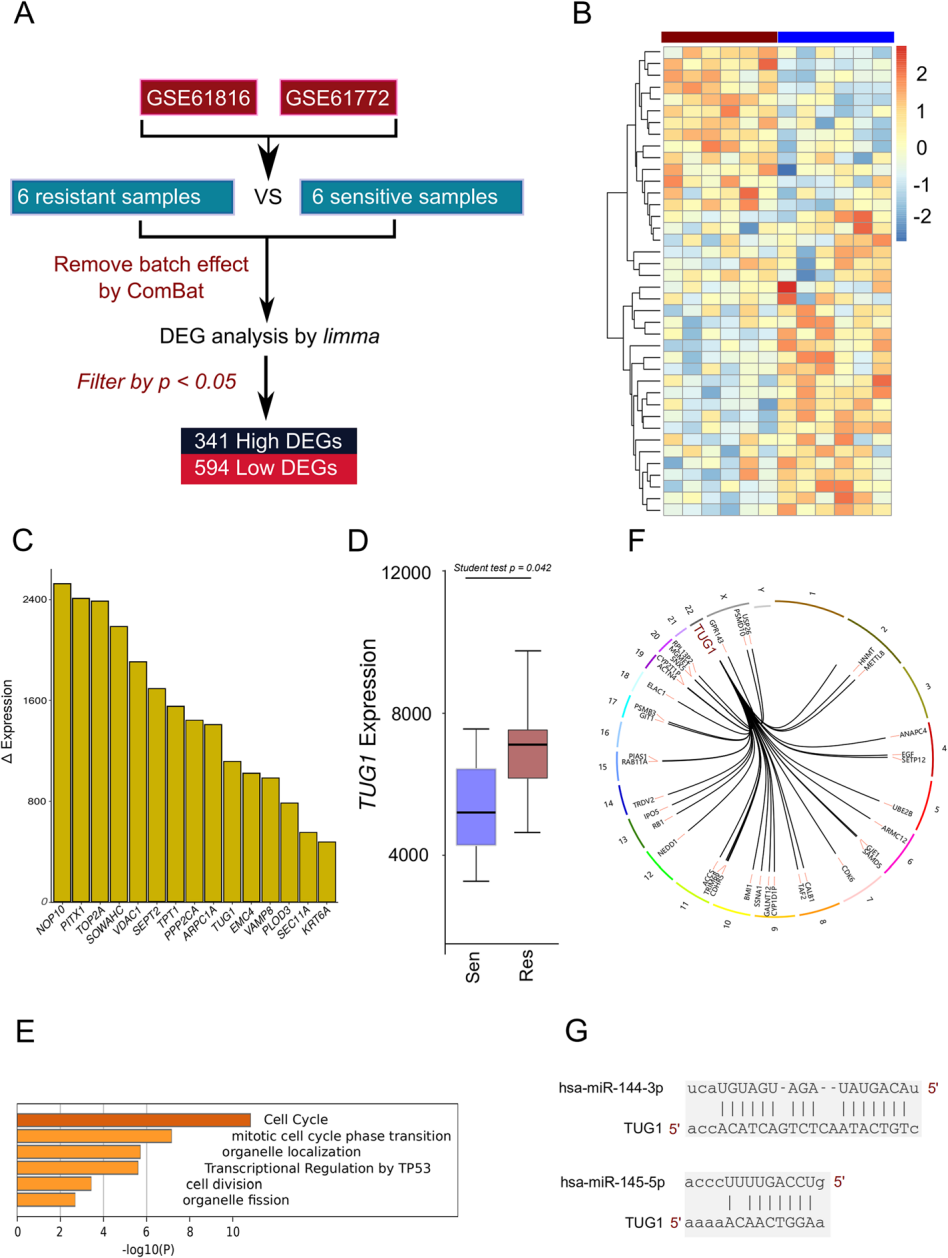
Bioinformatics analysis shows that lncTUG1 might participate in ESCC **(a**). The basic flow of data mining analysis; (**b**). The genes with significantly different expression are shown as a heat map, relative enrichment scores indicate upregulated (red) and downregulated (blue) genes across samples; (**c**). The top 15 genes with significantly higher expression in radioresistant cell lines; (**d**). The expression level of lncTUG1 in sensitive and resistant samples; (**e**). Pearson’s correlation of lncTUG1 and cell processes; (**f**). The Circos figure shows the genes from GO0044772 which are significantly correlated with lncTUG1; (**g**). The binding site between lncTUG1 and its potential interactors

To identify candidate genes that are associated with ESCC radioresistance, we performed a bioinformatics analysis using published expression data (Fig. 1a). Briefly, two data series consisting of two esophageal cancer cells and their derived radioresistant cell lines were obtained from the Gene Expression Omnibus (GEO) database (i.e., GSE61620, and GSE61772). Differential expression analysis was then performed between radioresistant and radiosensitive cell lines under different irradiation conditions using the normalized microarray data, which identified 341 genes that were significantly upregulated and 594 genes that were significantly downregulated in the radiosensitive cell lines compared with the radioresistant cell lines (*P* < 0.05; Fig. 1b). As shown in Fig. 1c and d, lncRNA-TUG1 was one of the most upregulated molecules, suggesting that it might play a role in the development of radiotherapy resistance in ESCC. To investigate the biological function of lncRNA-TUG1, genes whose expression levels were tightly correlated (absolute Pearson’s correlation coefficient value > 0.9) with that of the molecule in the cell lines were selected as the input for Metascape pathway analysis [26]. These genes were significantly enriched in meaningful cancer-related processes or pathways, such as the ‘Cell Cycle’ and ‘Transcriptional Regulation by TP53’ (Fig. 1e). To facilitate illustration, Circos was used to visualize the genes related to lncRNA-TUG1 expression in the GO0044772 term (Fig. 1f). To further explore the potential mechanism of lncTUG1 in radioresistance, RAID v2.0 was used to identify molecules that interact with lncTUG1 [27]. Indeed, we observed that hsa-miR-144-3p and hsa-miR-145-5p achieved the highest confidence scores among all types of interactors. The target prediction information is shown in Fig. 1g. Below, we focus on the relationship between lncTUG1 and hsa-miR-144-3p.

### lncTUG1 is upregulated in both ESCC tissues and cell lines

**Fig. 2:**
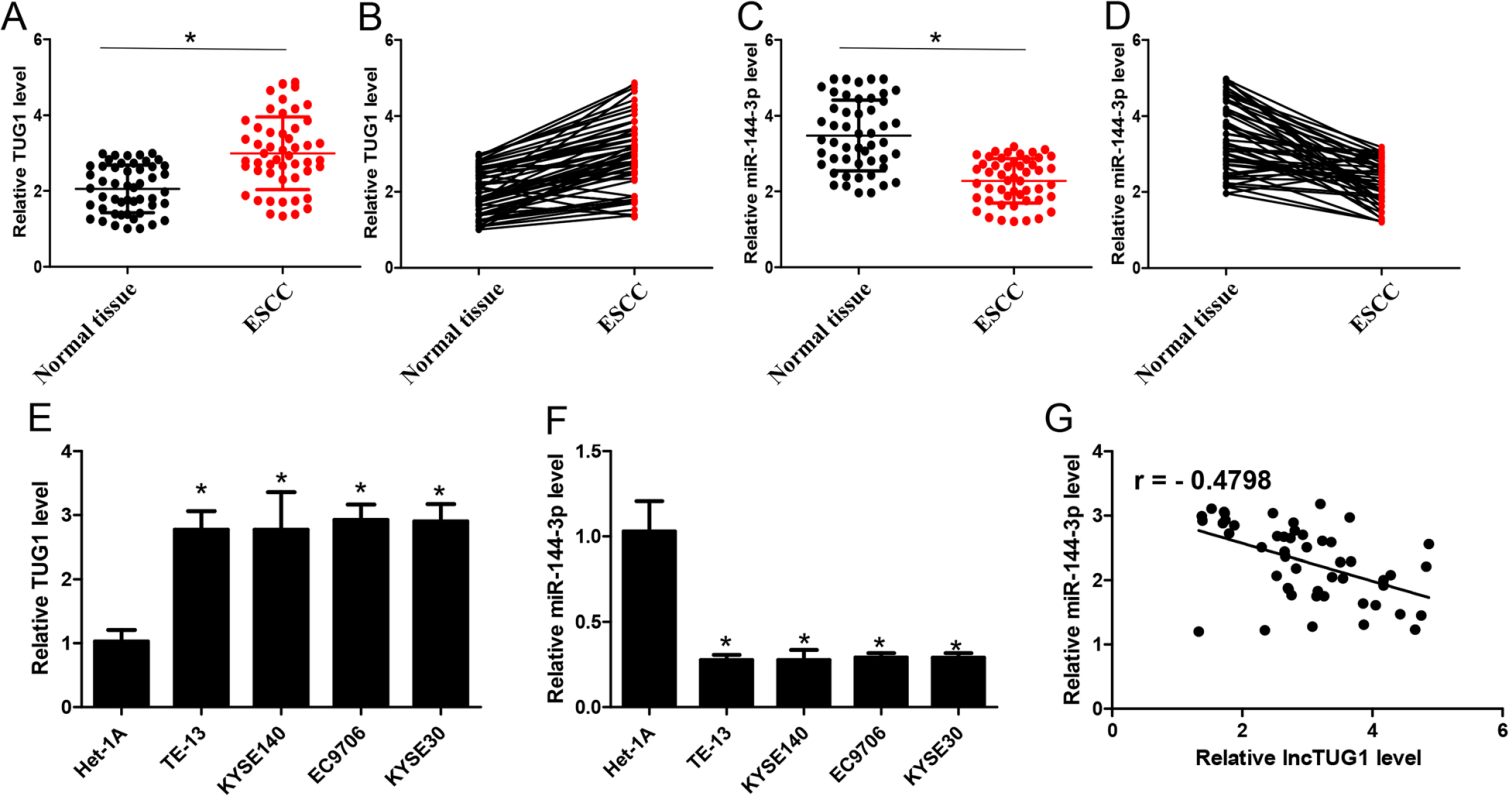
lncTUG1 is upregulated in ESCC tissues and cell lines. (**a**) and (**b**). The expression level of lncTUG1 in ESCC and matched adjacent normal esophageal tissues; (**c**) and (**d**). The expression level of miR-144-3p in ESCC and matched adjacent normal esophageal tissues; (**e**). The expression level of lncTUG1 in ESCC cell lines; (**f**) The expression level of miR-144-3p in ESCC cell lines; (**g**) The correlation between lncTUG1 and miR-144-3p. *P < 0.05

Based on the above results, we first examined the expression levels of lncTUG1 and its potential interacting miRNA, miR-144-3p, in ESCC and paired normal tissues. As shown in Fig. 2a-d, lncTUG1 was highly expressed in tumor tissues, while miR-144-3p was weakly expressed. In line with this result, the expression of lncTUG1 was increased in ESCC cell lines compared with normal esophageal cell lines (Fig. 2e). Moreover, decreased miR-144-3p expression was also observed in all the analyzed ESCC cell lines (Fig. 2f). Pearson’s correlation analysis confirmed that the expression of lncTUG1 was inversely correlated with that of miR-144-3p in both tissues and cell lines (Fig. 2g).

### lncTUG1 knockdown inhibits cell proliferation, migration and invasion

**Fig. 3:**
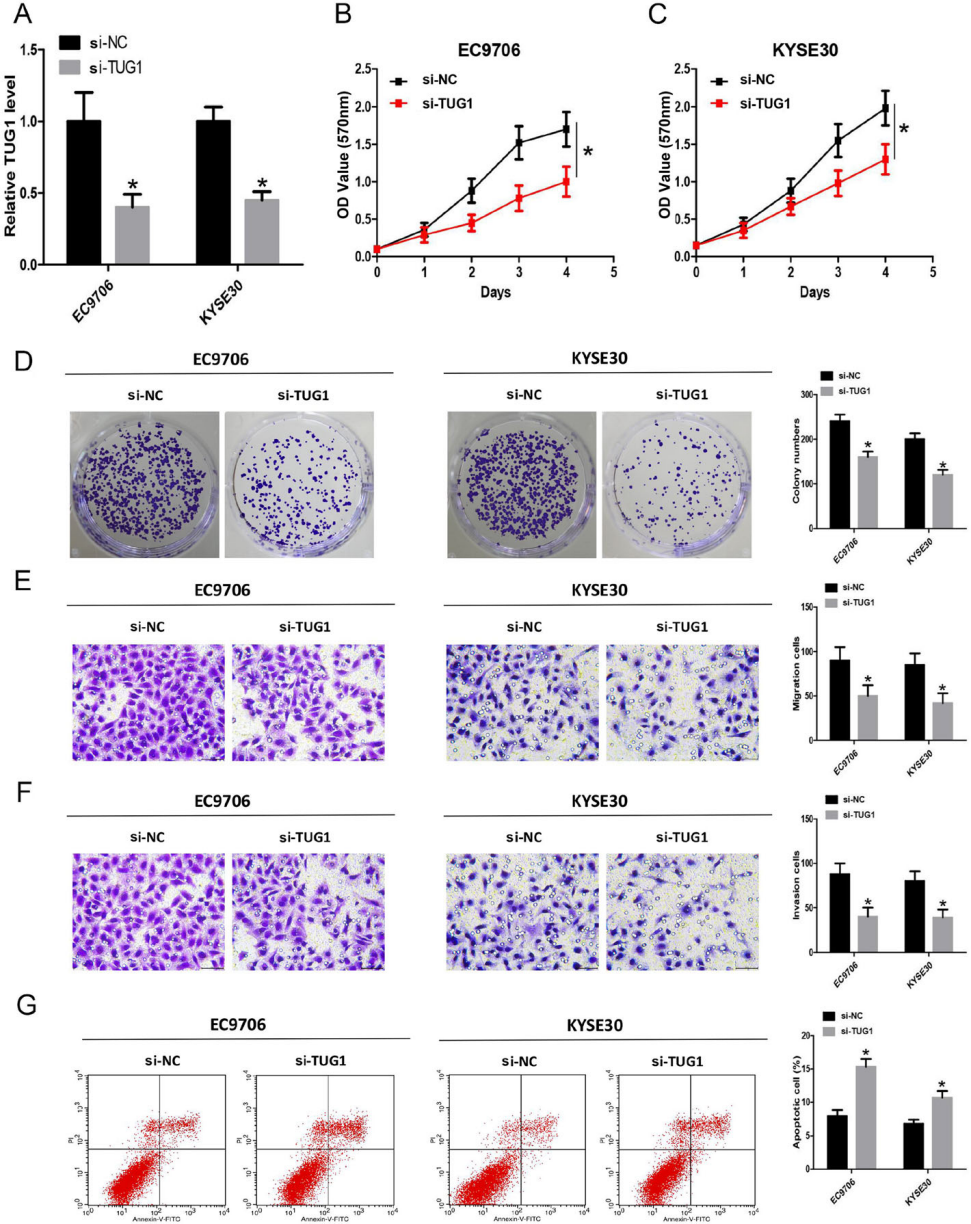
lncTUG1 knockdown inhibits ESCC cell proliferation, migration and invasion. **a** The level of lncTUG1 in EC9706 and KYSE30 cells; (**b**) and (**c**). Cell proliferation was evaluated with the MTT assay; (**d**). Cell proliferation was evaluated with the colony formation assay; (**e**) and (**f**). The migration and invasion abilities of EC9706 and KYSE30 cells; (**g**). Cell apoptosis was evaluated by flow cytometry. *P < 0.05

To investigate the functional role of lncTUG1 in tumorigenesis, we silenced lncTUG1 expression in EC9706 and KYSE30 cells with an siRNA. As shown in Fig. 3a, si-TUG1 was successfully transfected into the cell lines, and endogenous lncTUG1 was significantly suppressed. Then, the relative cellular abilities of proliferation, migration and invasion were examined. si-TUG1 retarded the growth of EC9706 and KYSE30 cells according to the MTT assay (Fig. 3b and c), and the results of the colony formation assay were largely consistent (Fig. 3d). Moreover, the low level of lncTUG1 expression led to a downward trend in both migration and invasion (Fig. 3e and f). In contrast, lncTUG1 knockdown increased the quantity of apoptotic cells (Fig. 3g). Taken together, these results indicate that lncTUG1 is a potential oncogenic factor that influences the progression of ESCC.

### lncTUG1 is involved in ESCC radiotherapy and affects radiosensitivity

**Fig. 4:**
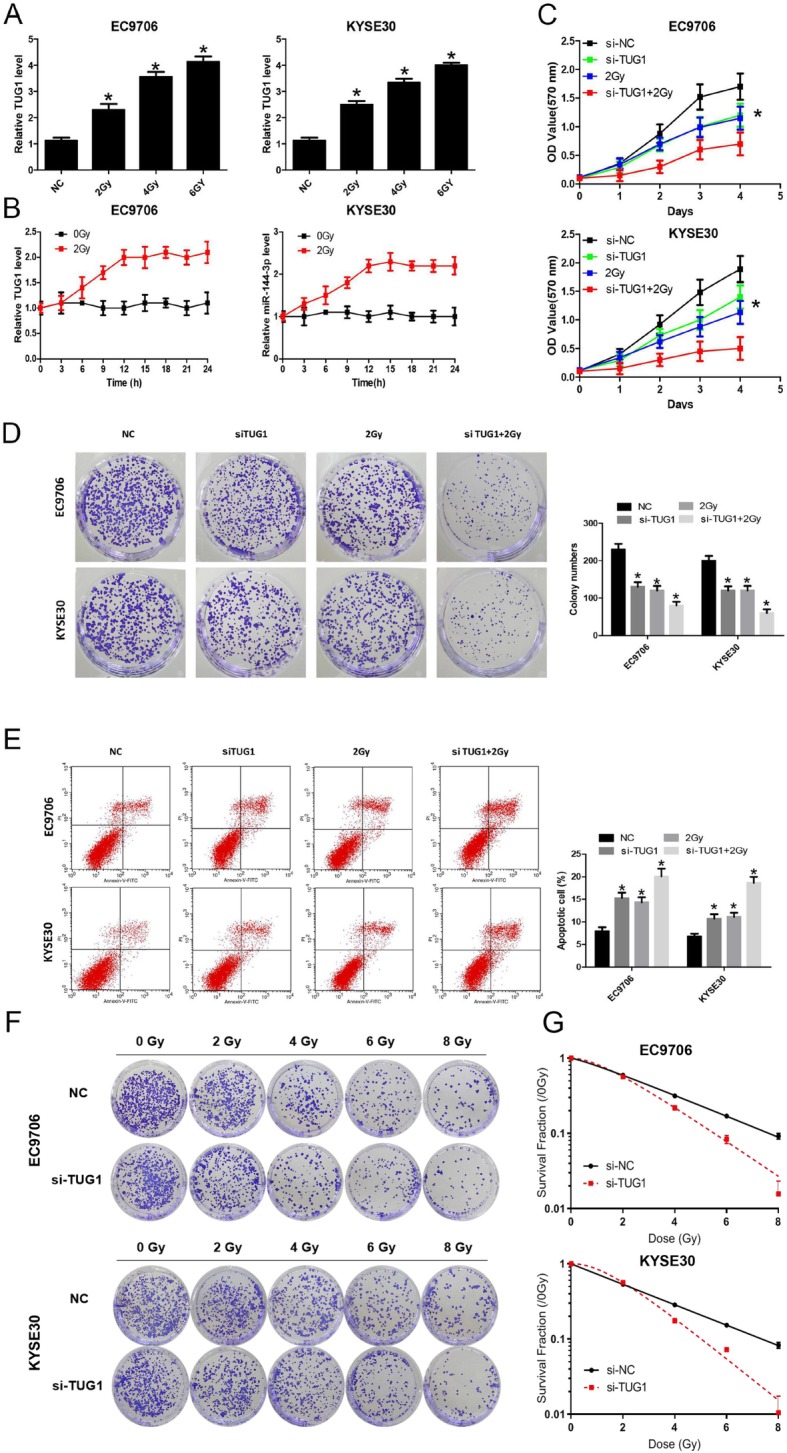
lncTUG1 is involved in ESCC radiotherapy and affects radiosensitivity. **a** and **b**. The level of lncTUG1 in EC9706 and KYSE30 cells; (**c**) and (**d**). Cell proliferation was evaluated with MTT and colony formation assays; (**e**). Cell apoptosis was evaluated by flow cytometry; (**f**). The colony forming ability of EC9706 and KYSE30 cells were exposed to 0, 2, 4, 6, 8 Gy; (**g**). the cellular survival curves of EC9706 and KYSE30 cells. *P < 0.05

Because the analysis was performed in the radiotherapy samples, the mechanism by which lncTUG1 affects the radiosensitivity of ESCC cells was the point of interest. The level of lncTUG1 was examined in a time- and dose-dependent manner in EC9706 and KYSE30 cells. Both the dose and time affected the expression level of lncTUG1 (*P < 0.05, Fig. 4a and b). More importantly, si-TUG1 combined with 2 Gy radiation showed increased radiation sensitivity in ESCC cells. The MTT assay indicated that this combined treatment had significant inhibitory effects on cell proliferation (*P < 0.05, Fig. 4c). EC9706 and KYSE30 cell colonies were inhibited dramatically by lncTUG1 knockdown plus 2 Gy radiation (Fig. 4d). Moreover, this combined treatment further induced the apoptosis of EC9706 and KYSE30 cells (*P < 0.05, Fig. 4e). Besides these results, in Fig. 4f, we conducted the colony formation assay of EC9706 and KYSE30 cells exposed to the different radiotherapy doses (0, 2, 4, 6, 8 Gy). As shown in Fig. 4g, the cellular survival curves of EC9706 and KYSE30 cells indicated the knockdown of lncTUG1 indeed enhance the radiosensitivity, and the relative relative radiosensitizaion effects data were shown in Table 3. All these results indicate that lncTUG1 is involved in ESCC radiotherapy and affects radiosensitivity.

**Table 3:** The relative radiosensitizaion effects in EC9706 and KYSE30

	D0 (Gy)	Dq (Gy)	N	SF_2_ (%)
EC9706				
si-NC	3.073 ± 0.124	1.475 ± 0.079	1.616 ± 0.032	69.6 ± 1.0
si-TUG1	1.865 ± 0.110*	1.297 ± 0.042	2.005 ± 0.093*	56.8 ± 0.9
KYSE30				
si-NC	3.209 ± 0.123	2.215 ± 0.124	1.994 ± 0.023	78.3 ± 0.9
si-TUG1	1.578 ± 0.114*	1.463 ± 0.039	2.528 ± 0.155*	56.7 ± 0.8

*P < 0.05

### lncTUG1 affects ESCC progression via the miR-144-3p/MET axis

**Fig. 5:**
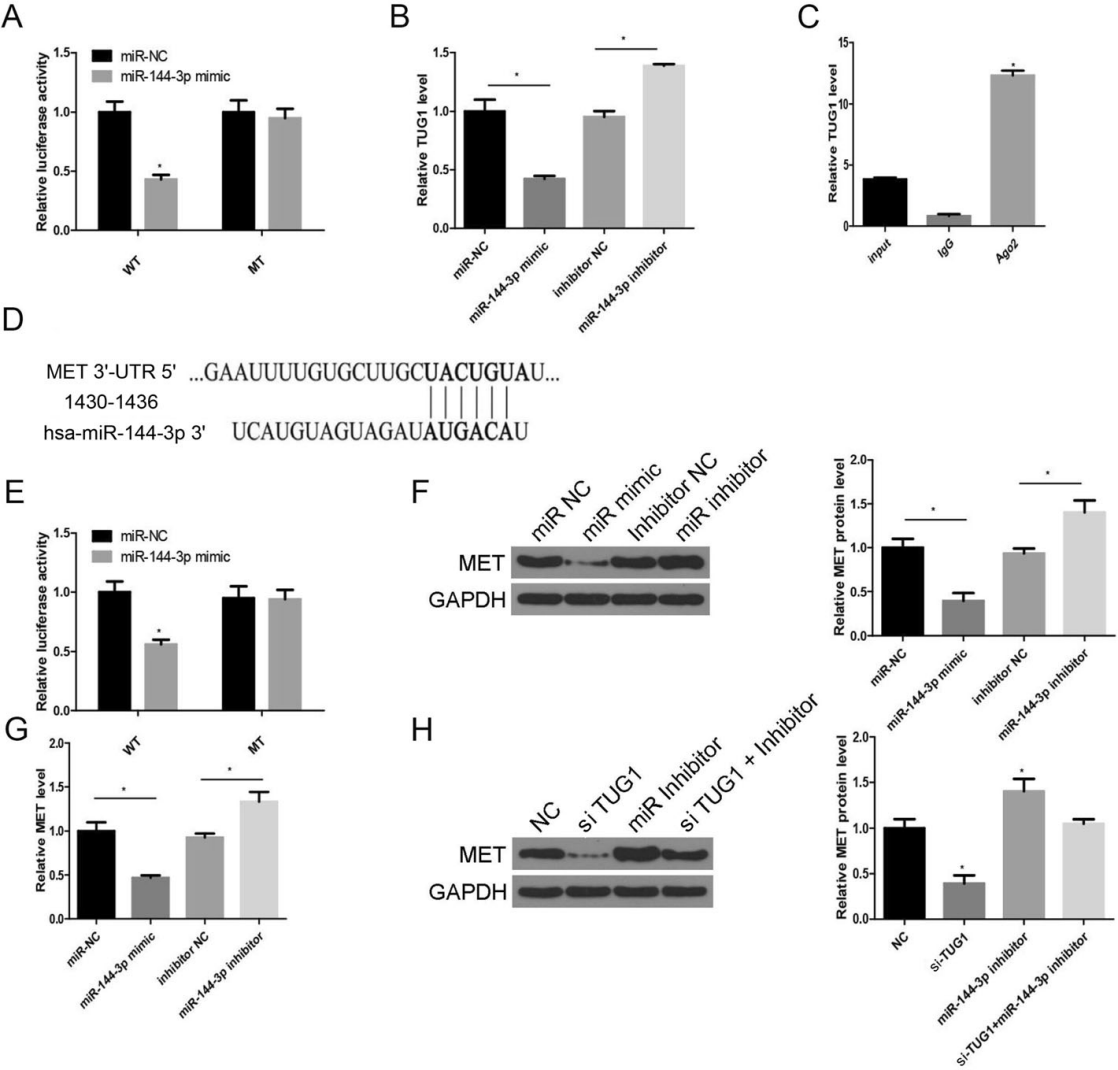
lncTUG1 affects ESCC progression via the miR-144-3p/MET axis. **a**. Luciferase activity was examined in HEK293T cells; (**b**). The level of lncTUG1 in KYSE30 cells; (**c**). The interaction between miR-144-3p and LncTUG1 was detected by RNA immunoprecipitation; (**d**). The potential binding site between miR-144-3p and MET; (**e**). Luciferase activity was examined in HEK293T cells; (**f**). MET protein level in KYSE30 cells; (**g**). MET mRNA level in KYSE30 cells; (**h**). MET protein level in KYSE30 cells. *P < 0.05

miR-144-3p was chosen as a target based on the previous prediction. The luciferase system indicated that only the miR-144-3p mimic decreased the luciferase activity of WT-TUG1 but had no effect on Mut-TUG1 (Fig. 5a). The lncTUG1 level was affected by the miR-144-3p level (Fig. 5b). Moreover, the RIP assay further confirmed that lncTUG1 was significantly augmented by Ago2 but not IgG (Fig. 5c). To further identify the target of hsa-miR-144-3p, the 3′-UTR of MET with the potential binding site was examined (Fig. 5d). The luciferase reporter system showed that the luciferase activity of the 3′-UTR of only WT MET was decreased (Fig. 5e). Both the protein and mRNA levels of MET were affected by the miR-144-3p level (Fig. 5f and g). Moreover, the miR-144-3p inhibitor reversed the si-TUG1 effect on the protein level of MET (Fig. 5h). Based on these results, we conclude that the lncTUG1/ miR-144-3p/MET axis indeed exists.

### The miR-144-3p inhibitor restores the effect of lncTUG1 knockdown on radiotherapy

**Fig. 6:**
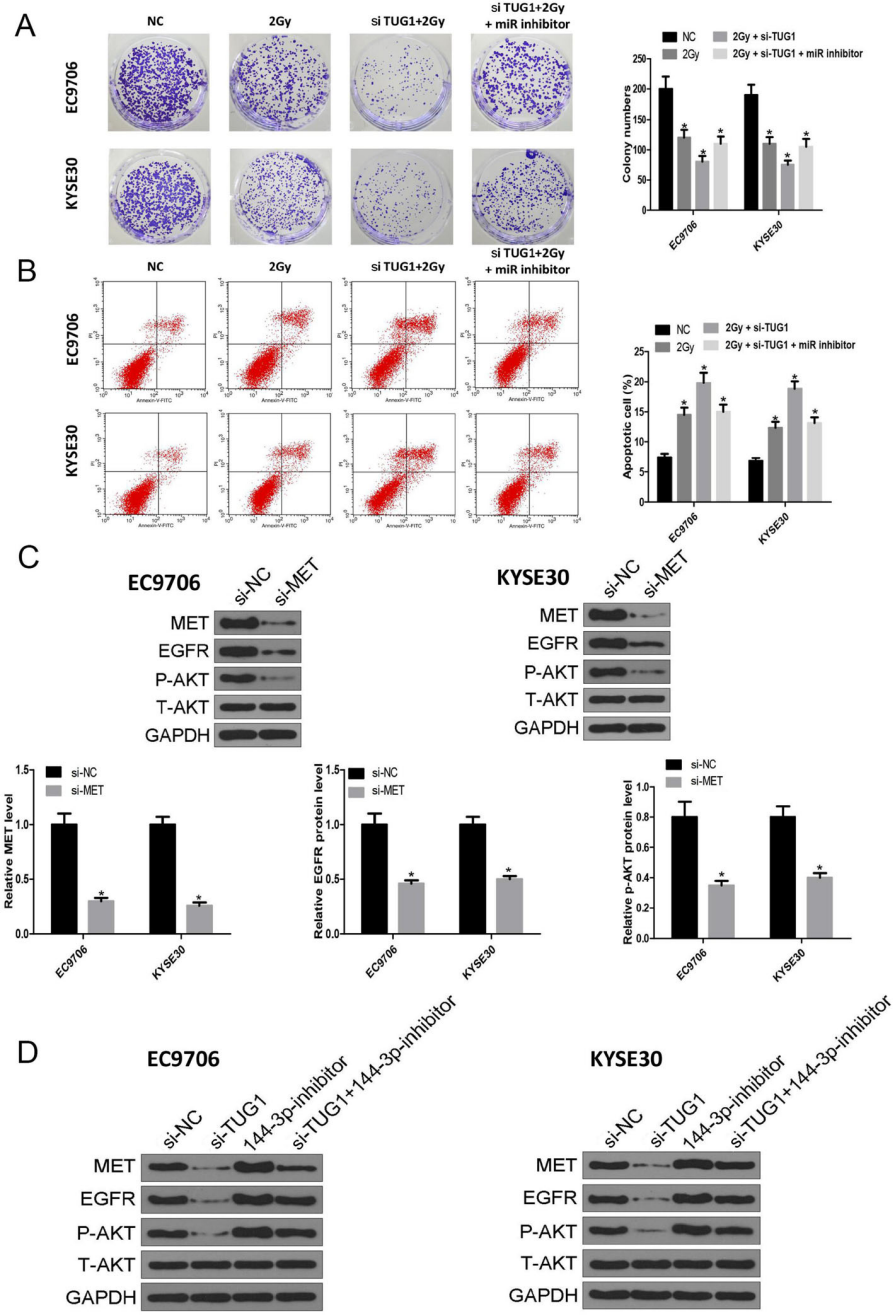
The miR-144-3p inhibitor restores the effect of lncTUG1 knockdown on radiotherapy. **a** Cell proliferation was evaluated by colony formation assays; (**b**). Cell apoptosis was evaluated by flow cytometry; (**c**) and (**d**). The protein levels of MET, p-AKT and t-AKT in EC9706 and KYSE30 cells. *P < 0.05

According to the above results, lncTUG1 affects ESCC progression via the miR-144-3p/MET axis. We further determined whether lncTUG1 affects radiosensitivity through miR-144-3p and MET. As shown in Fig. 6a and b, colony formation and apoptosis assays confirmed that the miR-144-3p inhibitor restored the effect of lncTUG1 knockdown on radiotherapy. Moreover, MET knockdown decreased the level of EGFR and lowered the phosphorylation level of AKT (Fig. 6c). It is possible that the p-AKT level is the key factor in ESCC radiotherapy.

### In vivo experiments confirmed that the inhibition of lncTUG1 enhances ESCC radiosensitivity

**Fig. 7:**
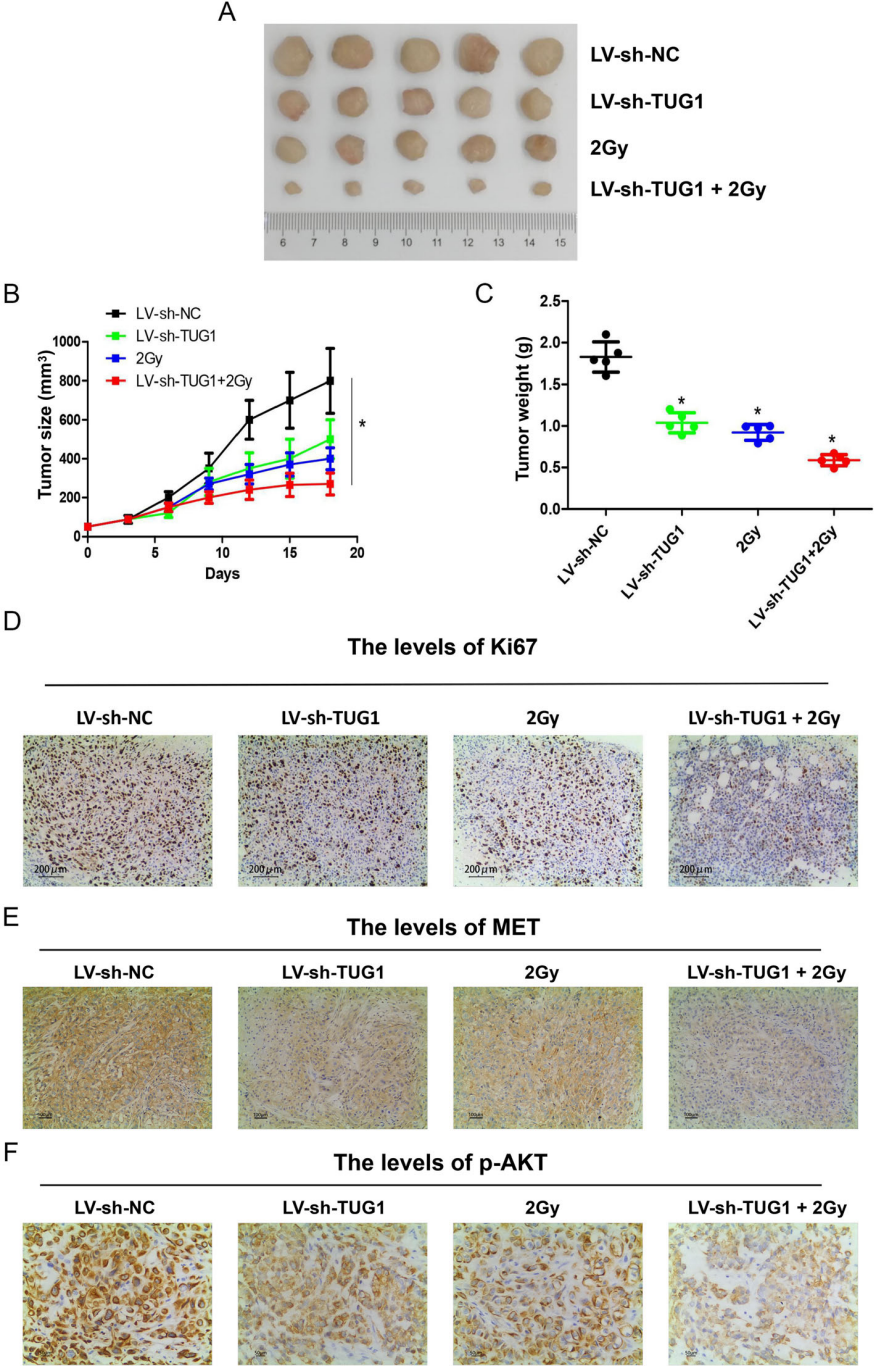
In vivo experiments confirmed that the inhibition of lncTUG1 enhances ESCC radiosensitivity. **a** Tumor images; (**b**) Tumor growth curves; (**c**). Tumor weights; (**d**), (**e**) and (**f**) Ki67, MET and p-AKT tumor levels based on IHC. *P < 0.05

Finally, we aimed to explore the effect of lncTUG1 on the radiosensitivity of ESCC tumor tissue. We subcutaneously injected transfected (LV-NC or LV-TUG1) KYSE30 cells into BALB/c nude mice to establish an in vivo model. The findings indicated that lncTUG1 knockdown could enhance the effect of radiotherapy on ESCC in vivo (Fig. 7a, b and c). All of these results exhibited the smallest tumor volume, the slowest tumor growth and the lightest tumor weight when the LV-TUG1 KYSE30 underwent 2 Gy radiation in this xenograft model. Meanwhile we also detected the downstream target expression. The tumor level of Ki67 was also reduced dramatically in the sh-TUG1 plus 2 Gy group (Fig. 7d). The tumor level of MET and p-AKT had the same trend that the lowest level of MET and p-AKT in the sh-TUG1 plus 2 Gy group (Fig. 7e and f).

## Discussion

In this study, we discovered that lncTUG1, as an oncogenic factor, participates in the progression of ESCC. More importantly, the role of lncTUG1 in the radiosensitivity of ESCC was investigated. Our findings revealed that lncTUG1 increases the expression of MET by sponging miR-144-3p and then activates the AKT signaling pathway to promote the progression of ESCC, including inhibiting apoptosis and inducing proliferation, migration and invasion. These results were consistent with those of previous reports that indicated that lncTUG1 might be an oncogenic factor. For example, Li Y et al. found that lncTUG1 was upregulated in renal cell carcinoma and acted as a miR-299-3p sponge to promote tumorigenesis by activating the VEGF pathway [28]. Xu T et al. also reported that lncTUG1 accelerated prostate cancer tumorigenesis and was associated with a poor prognosis [29]. Based on our findings, lncTUG1 promotes proliferation, migration and invasion but inhibits apoptosis in ESCC cells. In summary, we believe that lncTUG1 should serve as an oncogenic factor in the development of ESCC.

miR-144-3p and MET were found to affect the development of ESCC. Moreover, we verified that miR-144-3p can downregulate the expression of MET by the dual-luciferase reporter system. Mushtaq et al. reported that miR-144 exhibited tumor suppressive effects on gastric cancer cells [30]. It was reported that a high level of miR-144, as a promising therapeutic strategy, alleviated resistance to chemotherapy in glioblastoma cells [31]. miR-144-3p can inhibit the Src-Akt-Erk pathway to retard the progression of lung cancer [32]. Moreover, numerous studies have indicated that MET is associated with activation of the AKT signaling pathway by upregulating the expression level of EGFR. MET/EGFR signaling modulates cell proliferation in lung cancer [33]. The biological roles of these factors are consistent with our findings; thus, we provide new insights into the oncogenic role of lncTUG1, which promotes the development of ESCC through the miR-144-3p/MET/AKT axis.

We also note that there are some limitations to our study. Both lncTUG1 and miR-144-3p could have additional targets needed to exert their biological functions. They may play important roles in ESCC through multilevel regulation, leading to synthetic effects. By high-throughput sequencing analysis, the underlying biological changes in the different expression levels of lncTUG1 will be uncovered.

More importantly, we are highly concerned with improving the effect of radiotherapy on ESCC. First, by analyzing expression information on ESCC tissues and radiotherapy samples from the GEO database, we found an apparent difference in lncTUG1 between sensitive and resistant samples. Second, in combination with 2 Gy radiotherapy, we verified that lncTUG1 affected the progression of ESCC in vivo and in vitro. This result suggests that lncTUG1 regulates radiosensitivity in ESCC. Third, the phosphorylation of AKT, as a key factor related to radiosensitivity, is influenced by the level of lncTUG1. Notably, lncTUG1 exerts an apparent radiotherapy resistant effect on ESCC. Thus, lncTUG1 knockdown potentially has significant clinical application value.

## Conclusions

In conclusion, our study clarifies that lncTUG1 elevates the expression of MET by sponging miR-144-3p and then activates the AKT signaling pathway to affect the radiosensitivity of ESCC.

## Data Availability

The analyzed data sets generated during the study are available from the corresponding author on reasonable request.
